# Inhibitory effect of Astragalus Membranaceus on osteoporosis in SAMP6 mice by regulating vitaminD/FGF23/Klotho signaling pathway

**DOI:** 10.1080/21655979.2021.1946633

**Published:** 2021-07-24

**Authors:** Yihui Chai, Xiang Pu, Yongzhen Wu, Xingzhong Tian, Qian Li, Fanyong Zeng, Jing Wang, Jie Gao, Huaqian Gong, Yunzhi Chen

**Affiliations:** a Resource Institute for Chinese Ethnic Materia Medica, Guizhou University of Traditional Chinese Medicine, Guiyang, China; b College of Basic Medical, Guizhou University of Traditional Chinese Medicine, Guiyang, China; c College of graduate, Guizhou University of Traditional Chinese Medicine, Guiyang, China; d Department of Traditional Chinese Medicine, Zhangjiakou No.5 Hospital, Zhangjiakou, China; e Department of Urology Surgery, Dejiang Nation Hospital of Traditional Chinese Medicine, Tongren, China; f College of Pharmacy, Guizhou University of Traditional Chinese Medicine, Guiyang, China

**Keywords:** Astragalus membranaceus, spontaneous senile osteoporosis, SAMP6, VD

## Abstract

Spontaneous senile osteoporosis severely threatens the health of the senior population which has emerged as a severe issue for society. A SAMP6 mouse model was utilized to estimate the impact of intragastrically administered Astragalus Membranaceus (AR) on spontaneous senile osteoporosis. Bone mineral density (BMD) and bone microstructure were measured using Micro-CT; contents of calcium and phosphorus were determined with the colorimetric method; and gene and protein expressions of fibroblast growth factor 23 (FGF23), Klotho, Vitamin D receptor (VDR), CYP27B1 and CYP24A1 were detected using qPCR, Western blot and ELISA assays, respectively. The findings indicated that AR could improve the femoral BMD and bone microstructure, elevate the contents of calcium and phosphorus, and increase the expression of Klotho, VDR, and CYP27B1 whereas decreasing the expression of FGF23 and CYP24A1 in SAMP6 mice in a dose independent manner. The present study has demonstrated that AR can promote osteogenesis and alleviate osteoporosis. It is also expected to provide a new insight for the treatment of spontaneous senile osteoporosis and to serve as a research basis for AR application.

## Graphical Abstract

**Figure:**
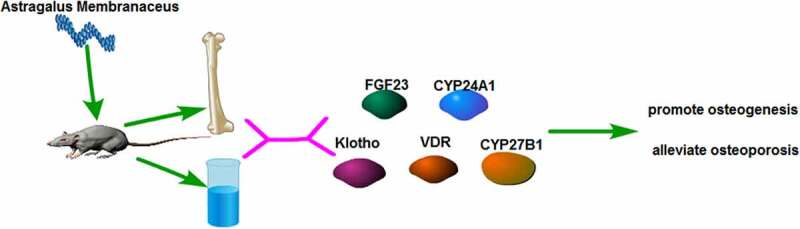

## Introduction

Spontaneous senile osteoporosis has been recognized as a degenerative skeletal disorder characterized by low bone mass, destruction of bone microstructure, increased bone brittleness, and prone to fracture as a result of aging [1,2]. Primary osteoporosis can be classified into postmenopausal osteoporosis (type **I**) which affects females and senile osteoporosis (type **II**) which affects both men and women [3,4]. Osteoporotic fractures are dangerous and the cause of high rates of disability and fatality. As the life span extends and the aging of the population, the number of osteoporotic patients has reached 110 million in the past four decades in China. Osteoporosis has been frequently encountered and posed a great threat to the health of the senior. The treatment and nursing of osteoporosis, osteoporotic fractures, and concomitant complications require tremendous labor, medical and financial resources. It becomes a heavy burden for families and society. Despite some progress that has been achieved recently, new strategies remain in urgent need for the prevention and treatment of the disease occurrence and development, thereby alleviating its clinical symptoms and improving patients’ quality of life.

VD, FGF23, and Klotho jointly regulate the metabolism of calcium and phosphorus within the body. Under physiological conditions, the precursor of vitamin D is hydroxylated twice by the liver and kidney, and the active 1,25(OH)2D3(OH)2D3 was generated under the action of kidney CYP27B, which plays an important physiological role by acting on diverse tissue cell VDR, including the human bones and heart [5,6].

Senescence accelerated mice were bred by a Japanese scholar in 1968 via inbreeding AKR/J mice. The animals have 14 strains of SAMP and 4 strains of senescence resistant mouse (SAMR). Accelerated senescence was observed in the P strain SAMP6 mice, manifestations including senile osteoporosis, systematic extensive osteopenia, bone tissue microstructure destruction, increased bone brittleness, low peak BMD and low peak bone mass. Some bone features of SAMP6 mice are similar to the manifestations of human senile osteoporosis [7,8]. For example, compared with the SAMR1, less bone mass was produced in the SAMP6 endosteum, fewer osteoblasts in the bone marrow, and lower bone strength, which can be used as a mouse model of senile osteoporosis. Since the SAMP6 mouse model of spontaneous senile osteoporosis is characterized by low peak bone mass, low BMD declined bone mass, and reduced osteoblast formation with age, it is the only animal used to prove brittle fracture of bones with age. Compared with other osteoporosis models, SAMP6 mice shows more advantages in the biological characteristics of bones that are identical to human beings; there are abundant experimental data available on the physiology, biochemistry, morphology, and pharmacology of SAMP6, which are helpful for research and comparison of the pathological mechanism of osteoporosis. Furthermore, the rapid reproduction of SAMP6 mice makes it possible to repeat multiple experiments due to the inbred lines with stable and uniform genetic information, little variations in bone anatomy, and physiological functions [9,10].

AR is a leguminous plant, collected from dried roots of Astragalus Mongolicus or Astragalus Membranaceus Bge. It is characterized by effects of tonifying qi, upraising yang, and invigorating wei for consolidating superficies. AR can also promote pus discharge and tissue regeneration as well as urination and alleviating edema. It has been widely reported that AR is effective in tonifying kidney qi directly in ancient literature. In *Compendium of Material Medical*, it says that ‘AR is sweet in odor, mildly warm and nontoxic. The main indications are for replenishing deficiency, treating pediatric diseases, tonifying deficiency for males and weakness from overwork, replenishing qi and benefiting yin, benefiting weakness, asthma, kidney failure, and deafness’. *Rihuazi Materia Medica* records that AR can ‘invigorate qi and strengthen muscles and bones’. Clinical applications of AR can increase lumbar BMD, inhibit bone resorption, maintain a positive balance by regulating bone metabolism and prevent bone loss in patients with primary osteoporosis. AR also can improve symptoms of postmenopausal osteoporosis, facilitate bone formation and restrict bone resorption. Cytological experiments have suggested that AR can markedly activate AKP, an important marker of osteoblast differentiation and maturation. It promotes the metabolism and protein synthesis of the induced BMSCs, enhances the proliferation of BMSCs and their differentiation into osteoblasts, elevates ALP activities, and inhibits the loss of bone collagen and bone phosphorus for osteoporosis prevention [11]. Similarly, AR indicates a role in elevating expression levels of Klotho gene to delay aging by regulating proteins related to cell cycles [12,13].

We hypothesized that AR could modulate the VD/FGF23/Klotho signaling pathway to alleviate osteoporosis. To clarify the regulatory effects of AR on spontaneous senile osteoporosis, the present study used SAMP6 mice administered intragastrically to detect BMD, bone microstructure, bone mineral contents, and the expression of related genes and proteins. This paper aims to investigate the intervention effects of AR on SAMP6 mice and is expected to provide a research basis for the treatment of spontaneous senile osteoporosis.

## Materials and methods

### Preparation of AR decoction

According to the requirements of *Chinese Pharmacopoeia* (2015 edition), qualified slices of AR were selected, and following the stipulated requirements and testing methods, the content of Astragaloside IV was determined using high performance liquid chromatography (HPLC) and those in compliance with the criteria were selected [14]. For HPLC, the column was Utrasphere-ODS 5 μm 4.6 mm*250 mm; the mobile phase was acetonitrile: 0.1 phosphoric acid (33:67); the flow rate was 1 ml/mim and the detection wavelength was 203 nm. Operation at room temperature.

An appropriate amount of dried AR was weighed and added in distilled water 10 times at the weight of AR, boiled 1 h with a gentle fire, immersed, and kept in water for 30 min. The liquid was filtered with gauze. Eight times of distilled water was subsequently added to the residue, boiled another 1 h, filtered the decoction, mixed both decoctions obtained, centrifuged, and concentrated the mixture with a rotary evaporator. The content of the crude decoction was at 1.0 g/mL.

### Animals

The animal experiments were performed following the management methods for laboratory animals of Guizhou University of Traditional Chinese Medicine and the requirements of the Ethics Committee of Guizhou University of Traditional Chinese Medicine. Twenty-five male SPF SAMP6 mice and 5 male SPF SAMR1 mice at 6 months of age, weighing 25 ± 5 g, were provided by Chongqing Ensville Biotechnology Co., Ltd. All animals were given adaptive feeding of one week and housed in conditions of 12 h light-dark cycles at 23–25°C. They had free access to diets and water.

The animals were divided into the following six groups: a SAMR1 group (normal saline), a SAMP6 group (normal saline), a SAMP6 + low dose AR group (2.4 g/kg), a SAMP6 + medium dose AR group (4.8 g/kg, a SAMP6 + high dose AR group (9.6 g/kg), and a SAMP6 + VD group (0.25 μg/d, 1, 25 (OH)2D3), five in each group and 30 in total.

The SAMP6 AR intervention groups were administered with high, medium, and low doses of the prepared AR decoction by gavage as per 10 μL/g of body weight, respectively. The SAMP6 VD intervention group was administered with a prepared VD solution by gavage as per 10 μL/g of body weight. The SMAR1 and SAMP6 control groups were given an equal volume of normal saline by gavage. The intervention lasted 12 weeks and the mice in each group were weighed once a week during an intervention. The dosage administered was adjusted according to the weight change of the mice [15]. Detailed body weight data during the experiment were presented in the attached ‘Body weight of the osteoporosis mice’.

### Sample collection

Samples were collected 12 h after the last administration.

Serum: Blood samples were collected through the eyeball after sacrifice of the mice, and centrifuged for 20 min at 3,000 g. The serum was separated and stored in a refrigerator at 4°C [16].

Femur: Long bones were separated and fixed with 4% paraformaldehyde. New bone formation (BV/TV) and BMD were detected by Micro-CT. Then decalcification was performed using 17% EDTA decalcification solution for 2 weeks, and the tissue sections were prepared for the subsequent staining experiments.

Determination of femur calcium and phosphorus contents: The femur was accurately weighed, baked at 600°C for 72 h until the weight was constant, then calcined in a muffle furnace at 800°C for 6 h, fully cooled and weighed the ash. Half of the bone ash was taken from each sample, and the contents of calcium and phosphorus were detected by test kits.

Isolation of bone marrow mesenchymal stem cells from the femur: The femur was cut to expose the bone marrow cavity. The bone marrow cavity of the mouse femur and tibia was rinsed repeatedly with PBS solution using a 1 ml syringe, flushed the bone marrow into a 1.5 ml EP tube, and centrifuged with 400 g for 5 min. The cells were resuscitated in a 6-well plate with 1 ml DMEM complete culture medium. The medium was refreshed every 2–3 days.

### Micro – CT assay

After 12 h of the last administration, all mice were anesthetized with 7% chloral hydrate and put to death. The long bones were taken, removed excess fascia and muscle tissues, and fixed and preserved with 4% paraformaldehyde for subsequent use.

Long bone samples were taken out from the 4% paraformaldehyde and kept in normal saline for 24 h. The whole femur, the proximal and intermediate parts were scanned using a Micro-CT detector. The proximal femur was scanned along the long axis at a scanning angle of 360 degrees and 8 μm resolution, and continuous planar micro-CT images were obtained. Thereafter, bone tissues at the 1.0 mm distal end of the growth version with 2.0 mm thickness were selected as the region of interest (ROI) on the host for three-dimensional reconstruction [17]. The lowest threshold was set at 160 for image extraction. After the capture of reconstructed images, quantitative analysis was made using the Micro-CT software. The main test parameters included the femoral BMD and cancellous bone parameter-bone volume fraction (BVF, BV/TV).

### Biomechanical detection of tissues in mice

12 h after the last administration, all mice were anesthetized with 7% chloral hydrate and put to death. Excess fascia and muscle tissues were removed completely. The three-point bending stress test of the femur was performed using an 858 MiniBionix material testing system for measurement and analysis. The loading speed was 5 mm/min with a 10 mm span. The biomechanical properties of the femur were tested including structural mechanical parameters of maximum load, elastic load, maximum winding, maximum bending moment, and energy absorption, and material mechanical parameters of maximum stress, elastic stress, rigidity coefficient, and elastic modulus [18].

### Real-time PCR

The isolation of total RNA from the cells was performed with TRIzol reagent (Invitrogen, USA) as per the instructions of the manufacturer. qRT-PCR was subsequently performed using a real-time PCR system (Bio-rad, USA) of the SYBR Green PCR Kit (Takara, Otsu, Japan) [19]. The expression of genes was normalized to β-actin. The primers used were listed as follows: VDR-F, 5ʹ-ACCCTGGTGACTTTGACCG-3ʹ; VDR-R, 5ʹ-CGGCAATCTCCATTGAAGGG-3ʹ; FGF23-F, 5ʹ-ATGCTAGGGACCTGCCTTAGA-3ʹ; FGF23-R, 5ʹ-AGCCAAGCAATGGGGAAGTG-3ʹ; CYP24A1-F, 5ʹ-CCTGAAGAAACAGCACGACAC-3ʹ; CYP24A1-R, 5ʹ-GTTGCGATGGTCCCGATA-3ʹ; Klotho-F, 5ʹ-GTGAGTCATTACACCACCATTC-3ʹ; BGP-F, 5ʹ-CCTGACTGCATTCTGCCTCT-3ʹ; BGP-R, 5ʹ-AGGTAGCGCCGGAGTCTATT-3ʹ; Klotho-R, 5ʹ-CATCGGGAGGTCTCCGTACT-3ʹ; CYP27B1-F, 5ʹ-TCCTGGCTGAACTCTTCTGC-3ʹ; CYP27B1-R, 5ʹ-GGCAACGTAAACTGTGCGAA-3ʹ; and β-Actin\-F, 5ʹ-GGCTGTATTCCCCTCCATCG-3ʹ; β-Actin-R, 5ʹ-CCAGTTGGTAACAATGCCATGT-3ʹ.

### Western blot analysis

Total protein extraction was performed using an ice-cold lysis buffer. Its quantification was subjected to the BCA kit (Beyotime, China). A quantity of 30 ng/well proteins was separated using SDS-PAGE gels and subsequently transferred into polyvinylidene difluoride (PVDF) membranes. Then the membranes were blocked using 5% skimmed milk at room temperature for 2 h and incubated with primary antibodies overnight at 4°C. Following three cycles of wash with TBST, the PVDF membranes were incubated with secondary antibodies at room temperature for 1.5–2 h, rinsed another three times with TBST, and then with TBS the last time [20]. An enhanced chemiluminescence detection system was used to visualize protein expression. Antibodies were listed in the following: FGF23, 1:500 (IGEE, BM6124); Klotho, 1:500 (IGEE, BM12028); VDR, 1:1000 (IGEE, BM2194); CYP27B1, 1:500 (IGEE, BM1716); CYP24A1, 1:500 (IGEE, BM1805); BGP, 1:500 (IGEE, BM11626); β-Actin, 1:2000 (IGEE, BMC026) and HRP Goat anti-Rabbit IgG (IGEE, BMS014).

### Detection of FGF23 and Klotho contents by ELISA

The contents of FGF23 (RD, DY2604-05) and Klotho (RD, DY5334-05) were detected according to the instructions of the ELISA kit. Briefly, an initial addition of 40 μL of sample diluent was supplemented to the sample wells in the enzyme coated plates, and 10 μL of samples to be tested were added. The samples were added to the bottom of the well in enzyme plates, vibrated gently, and mixed well, avoiding adhesion to the well wall. Incubation was performed at 37°C for 30 min following sealed with a sealing plate and repeated washing 5 times. 50 μL of enzyme reagent was added to each well and incubated at 37°C for 30 min. An addition of 50 μL chromogenic agent A was supplied in each well, followed by 50 μL chromogenic agent B, gently shook and mixed well for coloration at 37°C for 15 min in the dark. Subsequently, 50 μL of terminator was added to each well to discontinue the reaction. The absorbance of each well was measured sequentially at 450 nm wavelength [21].

### Statistical analyses

Data were expressed as mean with standard deviation (SD). Turkey’s tests were adopted for analysis of variance (ANOVA) for mean value comparison among all groups, and the P value less than 0.05 was considered statistically significant [22].

## Results

### AR increases bone density and bone microstructure in SAMP6 mice

We hypothesized that AR could modulate the VD/FGF23/Klotho signaling pathway to alleviate osteoporosis. To prove this hypothesis. We investigated the therapeutic effect of AR on osteoporosis using SAMP6 mice as experimental material and examined the gene and protein expression content of VD/FGF23/Klotho signaling pathway.

**Figure 1.**
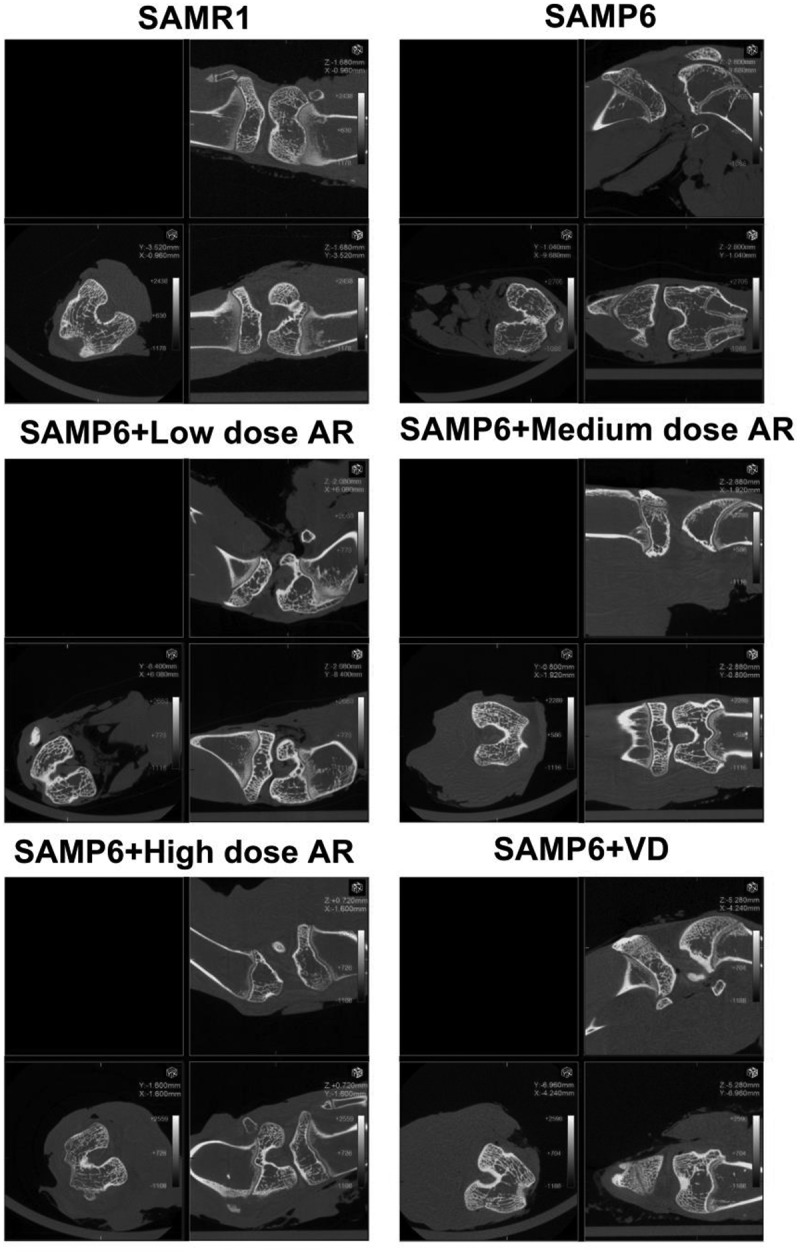
Detection of femur bone structure in mice by Micro-CT A-F showed in different treatment groups. A, SAMR1 group (normal control); B, SAMP6 group (negative control); C, SAMP6 + low dose AR group; D, SAMP6 + medium dose AR group; E, SAMP6 + high dose AR group; F, SAMP6 + VD (positive control)

**Figure 2.**
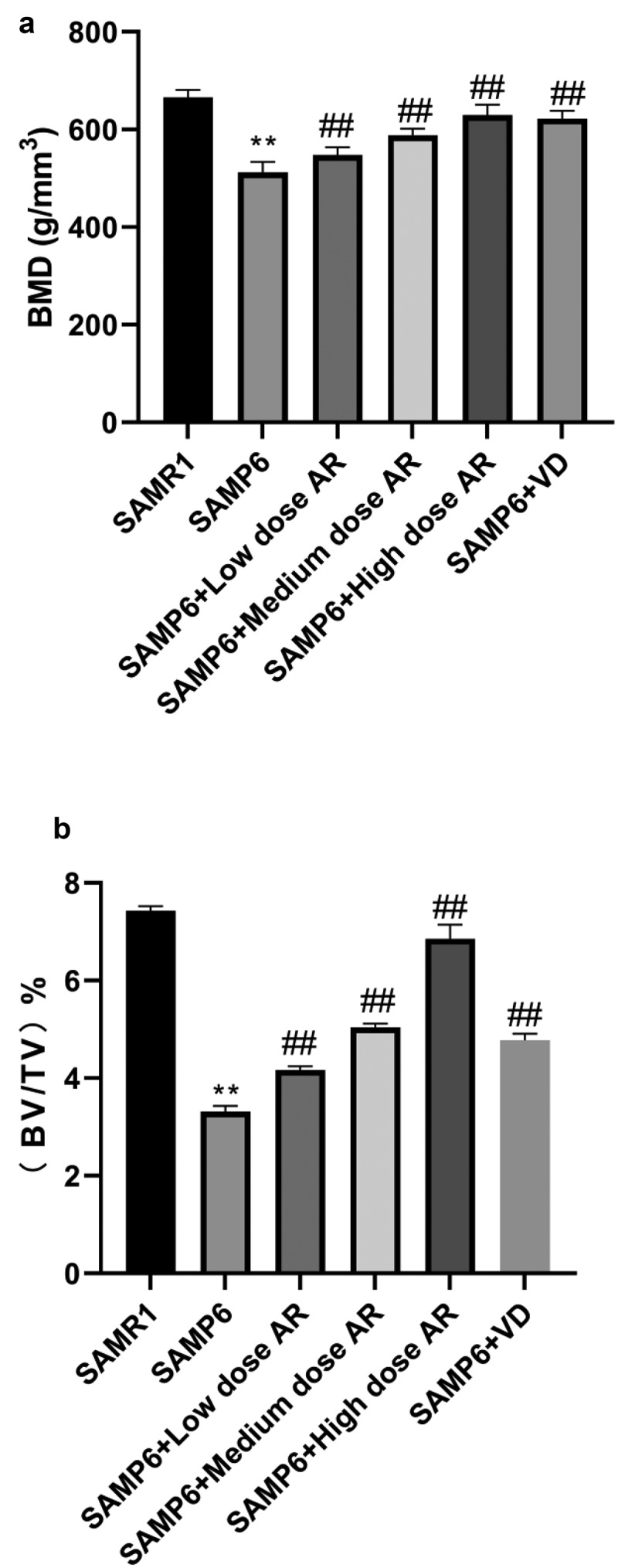
AR increases bone density and bone microstructure in SAMP6 mice. A, Bone mineral density test results of the SAMR1, SAMP6, SAMP6 + low dose AR, SAMP6 + medium dose AR, SAMP6 + high dose AR, and SAMP6 + VD groups; B, Bone microstructure test results of the SAMR1 group, SAMP6 group, SAMP6 + low dose AR group, SAMP6 + medium dose AR group, SAMP6 + high dose AR group, and SAMP6 + VD group. *P < 0.05, **P < 0.01, Compared with the normal group; #P < 0.05, ##P < 0.01, Compare with the model group. n = 5

Compared with the normal group three months later, the cancellous bone of the model group showed obvious loss of bone mass, sparse and thinning trabeculae, uneven arrangement with increased space, and destructed reticular structure. Meanwhile, large areas of no trabecular marrow were observed, including destructed bone microstructure and apparent thinning of the cortical bone layer. In the AR group, the bone trabeculae were thickened, the structures were compact with uniform distribution and thickness. The continuity was also good, and the reticular structure was nearly perfect. However, the thickness of cortical bone became thinner. After 6 months, the osteoporosis was aggravated in the model group over time. The bone trabeculae of the AR group became thinner and smaller, some were broken and unevenly arranged. The reticular structure began to be broken, and the bone marrow areas without bone trabeculae were observed. The cortical bone layer was thinner, developing a state of osteoporosis, but milder than the model group. As AR concentration increased, the protection and repair effects on bone structure were substantial (Figure 1). The BMD and bone microstructure data showed that BMD in the SAMR1, SAMP6, SAMP6 + low dose AR, SAMP6 + medium dose AR, SAMP6 + high dose AR and SAMP6 + VD groups were 660.24 ± 17.52, 546.36 ± 14.37, 582.70 ± 9.34, 628.71 ± 16.99, and 622.56 ± 21.66 g/mm^3^, respectively (Figure 2a), and the percentages of bone microstructure (BV/TV) were 7.41 ± 0.02, 4.14 ± 0.01, 4.96 ± 0.05, 6.82 ± 0.07, and 4.52 ± 0.03%, respectively (Figure 2b).

### Regulation of AR on structural mechanical parameters and material mechanical parameters of the femur

We conducted further detection of the structural mechanical parameters and material mechanical parameters of the femur, including maximum load, elastic load, maximum winding, maximum bending moment, energy absorption, maximum stress, elastic stress, rigidity coefficient, and elastic modulus. There were nine parameters in the SAMP6 groups that were lower than the SAMR1 group, whereas, after the administration of AR, the nine parameters increased markedly with the increase of AR dosage. The parameters of the high dose AR group were close to those in the SAMR1 group (Table 1 and Table 2).

**Table 1.** The structural mechanical parameters of the femur

Grouping	Maximum load (N)	Elastic load (N)	Maximum winding (mm)	Maximum bending moment (N˙mm)	Energy absorption (N˙mm)
SAMR1 group	22.34	18.61	1.61	44.02	18.46
SAMP6 group	12.39	10.21	1.28	26.36	8.57
SAMP6 + low-dose AR group	14.37	11.46	1.31	30.34	9.45
SAMP6 + medium-dose AR group	17.63	13.87	1.38	33.46	11.36
SAMP6 + high-dose AR group	19.56	16.53	1.52	39.76	14.89
SAMP6 + VD group	18.51	13.76	1.39	35.21	11.78

**Table 2.** The material mechanical parameters of the femur

Grouping	Maximum stress (N˙mm^2^)	Elastic stress (N˙mm^2^)	Rigidity coefficient (N˙mm^2^)	Elastic modulus (N˙mm^2^)
SAMR1 group	192.34	154.36	303.75	1142.66
SAMP6 group	112.41	100.06	192.36	1031.45
SAMP6 + low-dose AR group	118.63	112.34	224.68	1083.36
SAMP6 + medium-dose AR group	136.43	125.24	264.75	1101.75
SAMP6 + high-dose AR group	187.56	145.12	297.84	1156.58
SAMP6 + VD group	142.35	126.16	263.42	1121.47

### AR increases the contents of calcium and phosphorus in the femur of SAMP6 mice

Following the findings that AR could increase the BMD, bone microstructure, structural mechanical parameters, and material mechanical parameters of SAMP6 mice, a further investigation was conducted into the molecular regulatory mechanisms of AR.

**Figure 3.**
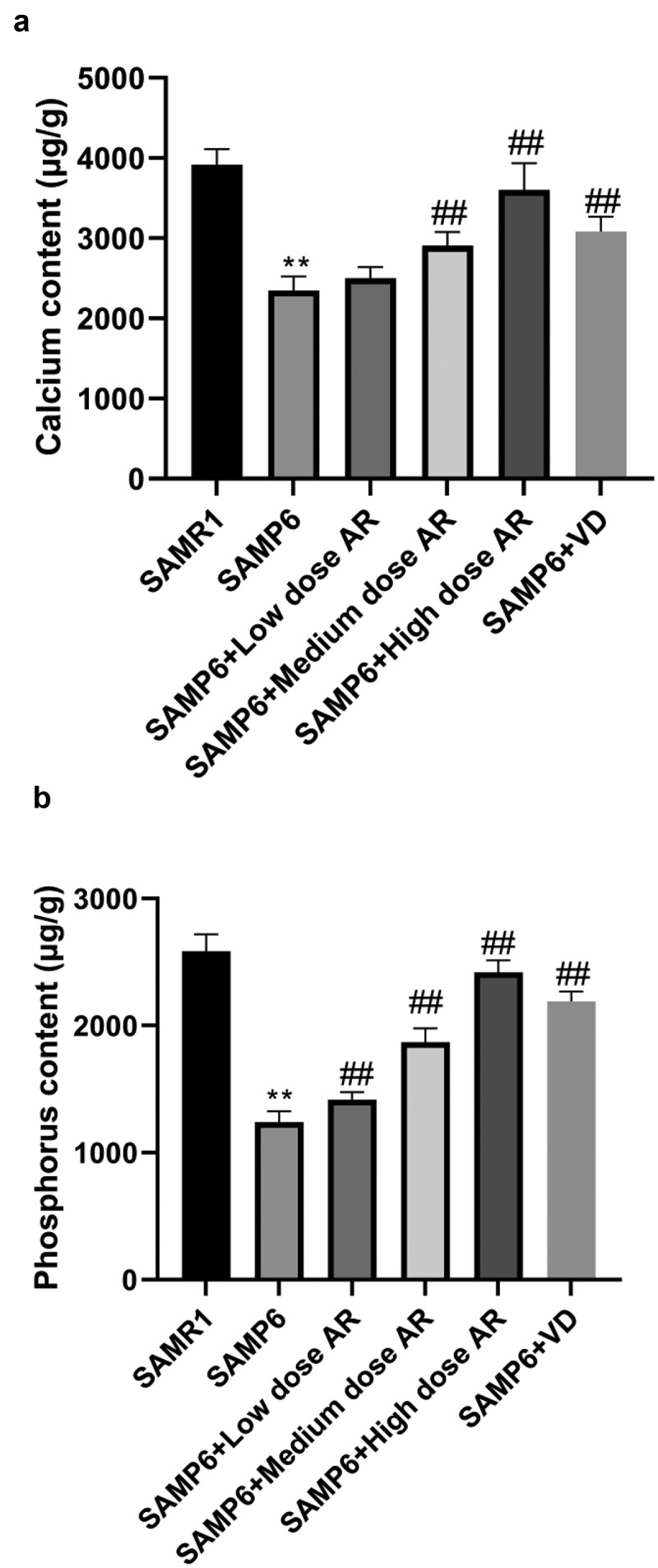
AR increases the contents of calcium and phosphorus in the femur of SAMP6 mice. A, Calcium content detection results of the SAMR1 group, SAMP6 group, SAMP6 + low dose AR group, SAMP6 + medium dose AR group, SAMP6 + high dose AR group, and SAMP6 + VD group; B, Phosphorus content detection results of the SAMR1 group, SAMP6 group, SAMP6 + low dose AR group, SAMP6 + medium dose AR group, SAMP6 + high dose AR group, and SAMP6 + VD group. *P < 0.05, **P < 0.01, Compared with the normal group; #P < 0.05, ##P < 0.01, Compare with the model group. n = 5

The contents of calcium and phosphorus in the femur were measured initially. The results indicated that the contents of calcium in the SAMR1, SAMP6, SAMP6 + low dose AR, SAMP6 + medium dose AR, SAMP6 + high dose AR, and SAMP6 + VD groups were 3915.26 ± 56.73, 2223.14 ± 4.72, 2347.25 ± 4.87, 2936.63 ± 25.68, 3836.24 ± 9.02, and 3249.40 ± 31.54 μg/g, respectively (Figure 3a). The contents of phosphorus were 2573.55 ± 10.60, 1426.72 ± 1.13, 1849.26 ± 38.34, 1849.26 ± 38.33, 2479.28 ± 11.03 and 2136.14 ± 10.88 μg/g, respectively (Figure 3b). The previously described results demonstrated that the contents of calcium and phosphorus in the femur of SAMP6 mice could be increased by AR intragastric administration.

### AR regulates the gene and protein expressions of FGF23, Klotho, VDR, CYP27B1, CYP24A1, and BGP

**Figure 4.**
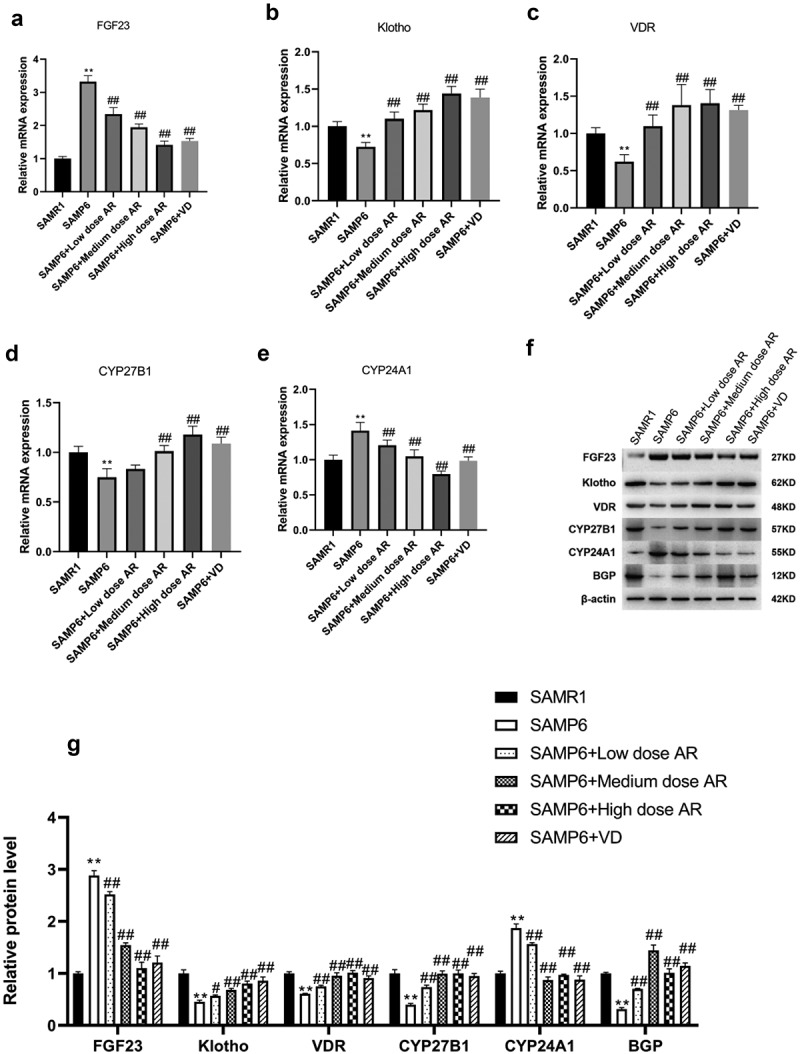
AR regulates the gene and protein expressions of FGF23, Klotho, VDR, CYP27B1, CYP24A1, and BGP. A-E, Detection of mRNA expression levels of FGF23, Klotho, VDR, CYP27B1, CYP24A1, and BGP by q-PCR; F, Detection of protein expression levels of FGF23, Klotho, VDR, CYP27B1, CYP24A1 and BGP by WB; G, Grayscale value of WB. *P < 0.05, **P < 0.01, Compared with the normal group; #P < 0.05, ##P < 0.01, Compared with the model group. n = 5

**Figure 5.**
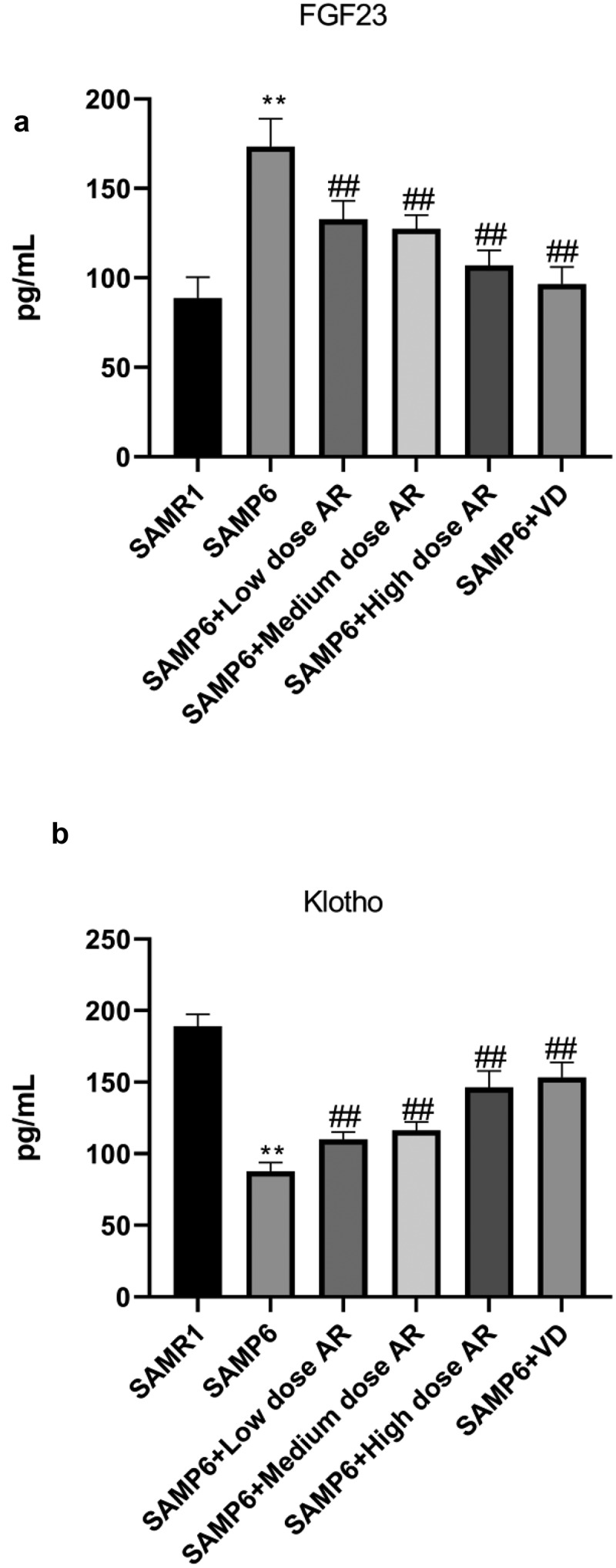
AR increases the content of FGF23 and Klotho. A, Detection of the expression of FGF23 in blood by ELISA; B, Detection of the expression of Klotho in blood by ELISA. *P < 0.05, **P < 0.01, Compared with the normal group; #P < 0.05, ##P < 0.01, Compared with the model group. n = 5

We ultimately detected the mRNA and protein expressions of FGF23, Klotho, VDR, CYP27B1, CYP24A1, and BGP. After the administration of AR by gavage, the mRNA and protein expressions of FGF23 and CYP24A1 in the BMSCs decreased in a dose-dependent manner, whereas the mRNA and protein expressions of Klotho, VDR, CYP27B1, and BGP in the BMSCs increased in a dose-dependent manner (Figure 4a-4g). Meanwhile, the expressions of FGF23 and Klotho in blood were detected by ELISA, and the results were consistent with qPCR and WB (Figure 5a-5b) indicating that AR could regulate VDR/FGF23/Klotho axis and affect the bone mass.

## Discussion

Osteoporosis is a degenerative disease of bone mass and bone microstructure. As it causes bones to be brittle, bone fractures occur most commonly [23]. It has developed as one of the primary diseases that seriously endangered the health of the gray population. The increasing incidence of fracture in osteoporosis patients is responsible for increasing catastrophic consequences namely disability and death caused by fractures. The density, elastic modulus and ultimate stress of cancellous bone decrease with age. Therefore, the associated changes in bone microstructure, density and biomechanics with age may lead to an increased risk of osteoporosis and fracture [20]. Bone mass and bone strength are determined by diverse comprehensive factors including heredity, nutrition, physical activity, and bone turnover [24]. The usual tests to assess bone metabolism include examination of serum calcium and phosphorus levels, as well as markers of bone formation and resorption. Microscopic CT analysis was used to examine the microstructure of trabecular and cortical bone. Dual-energy x-ray absorptiometry was used to detect bone mineral density [25]. In this study, we examined structural mechanical parameters and material mechanical parameters of the femur, and examined bone density and bone microstructure using microCT, and found that AR can significantly improve bone indexes and contribute to the recovery of osteoporotic symptoms.

VD is a hormone associated with changes in senile bone substance. VD deficiency is frequently found in the senior population globally. The reduced exposure to sunlight and declined intake of food rich in VD cause VD deficiency [26,27]. Moreover, as age increases, the VD metabolism of the skin also decreases. Secondary hyperparathyroidism usually stems from VD deficiency, which promotes osteoclastic bone resorption. In addition to hormonal changes, cytological alternations also occur in the bone microenvironment, including the movement and differentiation of mesenchymal stem cells, and subsequent changes in the structure of osteocytes [28]. The cell structure changes include increased adipocytes and decreased osteoblasts. Both osteoblasts and adipocytes have common progenitor cells in the bone marrow, so the increase in fat formation is at the expense of the decrease in osteoblast formation. Besides, apoptosis of more osteoblast will reduce the life cycle of osteoblasts. The cytological changes of aging bone reduce the number of osteoblasts available in bone remodeling and bone formation. Although the process of senile osteoporotic bone loss is the common result of the changes in hormone and bone cell structures, some cases of bone mass loss are just at the physiological level, while some are at the pathological level resulting from osteoporosis [29,30]. A SAMP6 mouse model of spontaneous senile osteoporosis was established in the present study and applied as experimental materials for investigating the regulatory effects of AR at different doses on osteoporosis administered by gavage. This study demonstrated that AR could increase the BMD, bone microstructure, and the contents of calcium and phosphorus in the femur of SAMP6 mice. As for the molecular mechanisms, we found that AR could regulate VD/FGF23/Klotho axis, increase the expression of VDR and Klotho, upregulate the expression of CYP27B1 and decrease the expression of CYP24A1. CYP27B1 acts as a protein promoting VD synthesis while CYP24A1 promotes the decomposition of VD. Furthermore, AR could upregulate the expression of CYP27B1 and decrease the expression of CYP24A1, which indirectly increased the synthesis of VD *in vivo*, promoted osteogenesis, and alleviated the symptoms of osteoporosis.

In our previous study, AR was added to bone marrow mesenchymal stem cells cultured *in vitro* to verify the regulatory effects of AR on the VD/FGF23/Klotho axis at the cellular level [12]. The results of the present study were consistent with our previous study at the animal level, which provided a novel idea for the treatment of spontaneous senile osteoporosis and a research foundation for the AR application in the future.

## Conclusion

The present study has demonstrated that AR can promote osteogenesis and alleviate osteoporosis. It is also expected to provide a new insight for the treatment of spontaneous senile osteoporosis and to serve as a research basis for AR application.

## Article highlight

AR improves the femoral BMD and bone microstructure.

AR promotes osteogenesis and alleviate osteoporosis.

AR increases the expression of Klotho, VDR, and CYP27B1 whereas decreases the expression of FGF23 and CYP24A1.

## Declarations

Research performed at Experimental Animals Laboratory, Guizhou University of Traditional Chinese Medicine, Guiyang, China.

## References

[1] Jesserer H. [Senile osteoporosis]. Zeitschrift Fur Alternsforschung. 1982;37(2):87–90. 7090435

[2] Riggs BL, Jowsey J, Kelly PJ, et al. Studies on pathogenesis and treatment in postmenopausal and senile osteoporosis. Clin Endocrinol Metab. 1973;2(2):317–332. 4373194 10.1016/s0300-595x(73)80046-5

[3] Qadir A, Liang S, Wu Z, et al. The involvement of differentiation and senescence of bone marrow stromal cells. Int J Mol Sci. 2020;21(1). 10.3390/ijms21010349 PMC6981793 31948061

[4] He J, Xu S, Zhang B, et al. Gut microbiota and metabolite alterations associated with reduced bone mineral density or bone metabolic indexes in postmenopausal osteoporosis. Aging (Albany NY). 2020;12(9):8583–8604. 32392181 10.18632/aging.103168 PMC7244073

[5] Haussler MR, Whitfield GK, Kaneko I, et al. The role of vitamin D in the FGF23, klotho, and phosphate bone-kidney endocrine axis. Rev Endocr Metab Disord. 2012;13(1):57–69. 21932165 10.1007/s11154-011-9199-8 PMC3288475

[6] Bover J, Urena-Torres P, Lloret MJ, et al. Integral pharmacological management of bone mineral disorders in chronic kidney disease (part II): from treatment of phosphate imbalance to control of PTH and prevention of progression of cardiovascular calcification. Expert Opin Pharmacother. 2016;17(10):1363–1373. 27156578 10.1080/14656566.2016.1182985

[7] Takada K, Inaba M, Ichioka N, et al. Treatment of senile osteoporosis in SAMP6 mice by intra-bone marrow injection of allogeneic bone marrow cells. Stem Cells. 2006;24(2):399–405. 16109754 10.1634/stemcells.2005-0068

[8] Ichioka N, Inaba M, Kushida T, et al. Prevention of senile osteoporosis in SAMP6 mice by intrabone marrow injection of allogeneic bone marrow cells. Stem Cells. 2002;20(6):542–551. 12456962 10.1634/stemcells.20-6-542

[9] Zhao X, Ai J, Mao H, et al. Effects of Eclipta prostrata on gut microbiota of SAMP6 mice with osteoporosis. J Med Microbiol. 2019;68(3):402–416. 30735116 10.1099/jmm.0.000936

[10] Ueda Y, Inui A, Mifune Y, et al. Molecular changes to tendons after collagenase-induced acute tendon injury in a senescence-accelerated mouse model. BMC Musculoskelet Disord. 2019;20(1):120. 30902076 10.1186/s12891-019-2488-1 PMC6429773

[11] Yang F, Yan G, Li Y, et al. Astragalus polysaccharide attenuated iron overload-induced dysfunction of mesenchymal stem cells via suppressing mitochondrial ROS. Cell Physiol Biochem. 2016;39(4):1369–1379. 27607448 10.1159/000447841

[12] Pu X, Chai Y, Guan L, et al. Astragalus improve aging Bone Marrow Mesenchymal Stem Cells (BMSCs) vitality and osteogenesis through VD-FGF23-Klotho axis. Int J Clin Exp Pathol. 2020;13(4):721–729. 32355520 PMC7191145

[13] Fu B, Yang J, Chen J, et al. Preventive effect of Shenkang injection against high glucose-induced senescence of renal tubular cells. Front Med. 2019;13(2):267–276. 29700792 10.1007/s11684-017-0586-8

[14] Guo LH, Cao Y, Zhuang RT, et al. IV promotes the proliferation and migration of osteoblast-like cells through the hedgehog signaling pathway. Int J Mol Med. 2019;43(2):830–838. 30535481 10.3892/ijmm.2018.4013 PMC6317662

[15] Yang B, Yang N, Chen Y, et al. An integrated strategy for effective-component discovery of astragali radix in the treatment of lung cancer. Front Pharmacol. 2020;11:580978. 33628171 10.3389/fphar.2020.580978 PMC7898675

[16] Liu W, Wang K, Lv X, et al. Up-regulation of RNA binding proteins contributes to folate deficiency-induced neural crest cells dysfunction. Int J Biol Sci. 2020;16(1):85–98. 31892848 10.7150/ijbs.33976 PMC6930370

[17] Shao B, Fu X, Yu Y, et al. Regulatory effects of miRNA181a on FasL expression in bone marrow mesenchymal stem cells and its effect on CD4+T lymphocyte apoptosis in estrogen deficiencyinduced osteoporosis. Mol Med Rep. 2018;18(1):920–930. 29845202 10.3892/mmr.2018.9026 PMC6059724

[18] Zhang Q, Zhao L, Shen Y, et al. Curculigoside protects against excess-iron-induced bone loss by attenuating Akt-FoxO1-dependent oxidative damage to mice and osteoblastic MC3T3-E1 cells. Oxid Med Cell Longev. 2019;2019:9281481. 31949885 10.1155/2019/9281481 PMC6948300

[19] Liu Y, Luo G, He D. Clinical importance of S100A9 in osteosarcoma development and as a diagnostic marker and therapeutic target. Bioengineered. 2019;10(1):133–141. 31055998 10.1080/21655979.2019.1607709 PMC6527076

[20] Shao H, Wu R, Cao L, et al. Trelagliptin stimulates osteoblastic differentiation by increasing runt-related transcription factor 2 (RUNX2): a therapeutic implication in osteoporosis. Bioengineered. 2021;12(1):960–968. 33734011 10.1080/21655979.2021.1900633 PMC8291811

[21] Yu C, Chen B, Zhao T, et al. High phosphate feeding induced arterial medial calcification in uremic rats: roles of Lanthanum carbonate on protecting vasculature. Life Sci. 2013;93(17):646–653. 24012609 10.1016/j.lfs.2013.08.013

[22] Zhang D, Yan K, Zhou J, et al. Myogenic differentiation of human amniotic mesenchymal cells and its tissue repair capacity on volumetric muscle loss. J Tissue Eng. 2019;10:1543350788. 10.1177/2041731419887100 PMC6851610 31762985

[23] Lorentzon M, Cummings SR. Osteoporosis: the evolution of a diagnosis. J Intern Med. 2015;277(6):650–661. 25832448 10.1111/joim.12369

[24] Yuan Y, Chen X, Zhang L, et al. The roles of exercise in bone remodeling and in prevention and treatment of osteoporosis. Prog Biophys Mol Biol. 2016;122(2):122–130. 26657214 10.1016/j.pbiomolbio.2015.11.005

[25] Xia J, Zhang Z, Wang J, et al. Comparison of the effects of heparin and the direct factor Xa inhibitor, rivaroxaban, on bone microstructure and metabolism in adult rats. Connect Tissue Res. 2015;56(6):477–482. 26305919 10.3109/03008207.2015.1069285

[26] Glowka E, Stasiak J, Lulek J. Drug delivery systems for Vitamin D supplementation and therapy. Pharmaceutics. 2019;11(7). 10.3390/pharmaceutics11070347 PMC6680748 31323777

[27] Liao EY, Zhang ZL, Xia WB, et al. (25-hydroxyvitamin D) improvement and calcium-phosphate metabolism of alendronate sodium/vitamin D3 combination in Chinese women with postmenopausal osteoporosis: a post hoc efficacy analysis and safety reappraisal. BMC Musculoskelet Disord. 2018;19(1):210. 29970059 10.1186/s12891-018-2090-y PMC6030763

[28] An ZM, Huang MJ, Zhang M, et al. [Relationship of 25(OH)VD with bone mass and other indicators in male patients with diabetes mellitus]. Sichuan Da Xue Xue Bao Yi Xue Ban. 2009;40(1):52–54. 19292044

[29] Zhao L, Li M, Sun H. Effects of dietary calcium to available phosphorus ratios on bone metabolism and osteoclast activity of the OPG /RANK/RANKL signalling pathway in piglets. J Anim Physiol Anim Nutr (Berl). 2019;103(4):1224–1232. 31062421 10.1111/jpn.13115

[30] Wang Y, Santiago FR, Deng M, et al. Identifying osteoporotic vertebral endplate and cortex fractures. Quant Imaging Med Surg. 2017;7(5):555–591. 29184768 10.21037/qims.2017.10.05 PMC5682396

